# Longitudinal Cognitive Changes in Genetic Frontotemporal Dementia Within the GENFI Cohort

**DOI:** 10.1212/WNL.0000000000200384

**Published:** 2022-07-19

**Authors:** Jackie M. Poos, Amy MacDougall, Esther van den Berg, Lize C. Jiskoot, Janne M. Papma, Emma L. van der Ende, Harro Seelaar, Lucy L. Russell, Georgia Peakman, Rhian Convery, Yolande A.L. Pijnenburg, Fermin Moreno, Raquel Sanchez-Valle, Barbara Borroni, Robert Laforce, Marie-Claire Doré, Mario Masellis, Maria Carmela Tartaglia, Caroline Graff, Daniela Galimberti, James B. Rowe, Elizabeth Finger, Matthis Synofzik, Rik Vandenberghe, Alexandre Mendonça, Pietro Tiraboschi, Isabel Santana, Simon Ducharme, Christopher Butler, Alexander Gerhard, Johannes Levin, Adrian Danek, Markus Otto, Isabelle Le Ber, Florence Pasquier, John van Swieten, Jonathan D. Rohrer, on behalf of the Genetic FTD Initiative (GENFI)

**Affiliations:** From the Department of Neurology (J.M. Poos, E.v.d.B., L.C.J., J.M. Papma, E.L.v.d.E., H.S., J.v.S.), Erasmus MC University Medical Center, Rotterdam, the Netherlands; Dementia Research Centre (J.M. Poos, L.C.J., L.L.R., G.P., R.C., J.D.R.), Department of Neurodegenerative Disease, UCL Institute of Neurology; Department of Medical Statistics (A.M.), London School of Hygiene and Tropical Medicine, UK; Department of Neurology (Y.A.L.P.), Alzheimer Center, Amsterdam University Medical Center, Amsterdam Neuroscience, the Netherlands; Cognitive Disorders Unit (F.M.), Department of Neurology, Donostia University Hospital, San Sebastian, Gipuzkoa; Alzheimer's Disease and Other Cognitive Disorders Unit (R.S.-V.), Neurology Service, Hospital Clínic, Institut d'Investigacións Biomèdiques August Pi I Sunyer, University of Barcelona, Spain; Centre for Neurodegenerative Disorders (B.B.), Neurology Unit, Department of Clinical and Experimental Sciences, University of Brescia, Italy; Clinique Interdisciplinaire de Mémoire (R.L., M.-C.D.), Département des Sciences Neurologiques, Université Laval, Québec; Sunnybrook Health Sciences Centre (M.M.), Sunnybrook Research Institute and Tanz Centre for Research in Neurodegenerative Diseases (M.C.T.), University of Toronto, Ontario, Canada; Department of Geriatric Medicine (C.G.), Karolinska University Hospital-Huddinge, Stockholm, Sweden; Centro Dino Ferrari (D.G.), University of Milan; Fondazione IRCCS Ca' Granda (D.G.), Ospedale Policlinico, Neurodegenerative Diseases Unit, Milan, Italy; Department of Clinical Neurosciences (J.B.R.), University of Cambridge, UK; Department of Clinical Neurological Sciences (E.F.), University of Western Ontario, London, Canada; Department of Neurodegenerative Diseases (M.S.), Hertie-Institute for Clinical Brain Research and Center of Neurology, University of Tübingen; German Center for Neurodegenerative Diseases (DZNE) (M.S.), Tübingen, Germany; Laboratory for Cognitive Neurology (R.V.), Department of Neurosciences, KU Leuven, Belgium; Faculty of Medicine (A.M.), University of Lisbon, Portugal; Fondazione Istituto di Ricovero e Cura a Carattere Scientifico Istituto Neurologica Carlo Besta (P.T.), Milan, Italy; Faculty of Medicine (I.S.), University of Coimbra, Portugal; Department of Psychiatry (S.D.), McGill University Health Centre, McGill University, Montreal, Québec, Canada; Department of Clinical Neurology (C.B.), University of Oxford; Divison of Neuroscience & Experimental Psychology (A.G.), Faculty of Medicine, Biology and Health, University of Manchester, UK; Departments of Geriatric Medicine and Nuclear Medicine (A.G.), Essen University Hospital, Germany; Department of Neurology (J.L., A.D.), Ludwig-Maximilians-University, Munich; German Center for Neurodegenerative Diseases (DZNE) (J.L.), Munich; Munich Cluster for Systems Neurology (SyNergy) (J.L.); Department of Neurology (M.O.), University of Ulm, Germany; Sorbonne Université (I.L.B.), Paris Brain Institute–Institut du Cerveau–ICM, Inserm U1127, CNRS UMR 7225, AP-HP–Hôpital Pitié-Salpêtrière; Centre de Référence des Démences Rares ou Précoces (I.L.B.), IM2A, Département de Neurologie, AP-HP–Hôpital Pitié-Salpêtrière; Univ Lille (F.P.); Inserm 1172 (F.P.); and CHU (F.P.), CNR-MAJ, Labex Distalz, LiCEND, Lille, France.

## Abstract

**Background and Objectives:**

Disease-modifying therapeutic trials for genetic frontotemporal dementia (FTD) are underway, but sensitive cognitive outcome measures are lacking. The aim of this study was to identify such cognitive tests in early stage FTD by investigating cognitive decline in a large cohort of genetic FTD pathogenic variant carriers and by investigating whether gene-specific differences are moderated by disease stage (asymptomatic, prodromal, and symptomatic).

**Methods:**

*C9orf72*, *GRN*, and *MAPT* pathogenic variant carriers as well as controls underwent a yearly neuropsychological assessment covering 8 cognitive domains as part of the Genetic FTD Initiative, a prospective multicenter cohort study. Pathogenic variant carriers were stratified according to disease stage using the global Clinical Dementia Rating (CDR) plus National Alzheimer's Coordinating Center (NACC) FTLD score (0, 0.5, or ≥1). Linear mixed-effects models were used to investigate differences between genetic groups and disease stages as well as the 3-way interaction between time, genetic group, and disease stage.

**Results:**

A total of 207 *C9orf72*, 206 *GRN*, and 86 *MAPT* pathogenic variant carriers and 255 controls were included. *C9orf72* pathogenic variant carriers performed lower on attention, executive function, and verbal fluency from CDR plus NACC FTLD 0 onwards, with relatively minimal decline over time regardless of the CDR plus NACC FTLD score (i.e., disease progression). The cognitive profile in *MAPT* pathogenic variant carriers was characterized by lower memory performance at CDR plus NACC FTLD 0.5, with decline over time in language from the CDR plus NACC FTLD 0.5 stage onwards, and executive dysfunction rapidly developing at CDR plus NACC FTLD ≥1. *GRN* pathogenic variant carriers declined on verbal fluency and visuoconstruction in the CDR plus NACC FTLD 0.5 stage, with progressive decline in other cognitive domains starting at CDR plus NACC FTLD ≥1.

**Discussion:**

We confirmed cognitive decline in the asymptomatic and prodromal stage of genetic FTD. Specifically, tests for attention, executive function, language, and memory showed clear differences between genetic groups and controls at baseline, but the speed of change over time differed depending on genetic group and disease stage. This confirms the value of neuropsychological assessment in tracking clinical onset and progression and could inform clinical trials in selecting sensitive end points for measuring treatment effects as well as characterizing the best time window for starting treatment.

Frontotemporal dementia (FTD) is a common cause of dementia, often presenting at a young age, with devastating effects on daily living.^1^ The typical cause of FTD is neurodegeneration of the frontal and temporal lobes resulting in behavioral disturbances (behavioral variant FTD [bvFTD]) or language impairment (primary progressive aphasia [PPA]).^2,3^ FTD is highly heritable and is autosomal dominantly inherited in up to ∼30% of cases. The most common causes are pathogenic variants in the microtubule-associated protein tau (*MAPT*), progranulin (*GRN*), or chromosome 9 open reading frame 72 (*C9orf72*) genes.^4^ Deficits in executive function, language, and social cognition are often predominant, but may vary in severity and progression due to the heterogeneous nature of the disease.^1-3,5^

Research into genetic FTD has shown that disease pathology emerges years before symptom onset.^6-13^ Initiating disease-modifying interventions at this early stage of the disease may have the most profound effect because neuronal loss is minimal and cognitive functions are preserved.^14^ It is therefore important to identify sensitive clinical instruments that can signal disease onset and track disease progression. Identifying such instruments for this early stage of the disease is also important because they can be used as clinical end points in therapeutic trials.

Gene-specific cognitive decline during the presymptomatic period has been demonstrated by both cross-sectional and longitudinal studies.^6,10,15-26^ For example, previous reports have shown decline in memory,^17,19,20,26^ language,^17,20,23^ and social cognition^17,19,20^ in *MAPT* pathogenic variant carriers, decline in attention^15,16,19,20^ and executive function^15,16,18,20^ in *GRN* pathogenic variant carriers, and a decline in social cognition in *C9orf72* pathogenic variant carriers.^22,24,25^ However, other studies on genetic FTD failed to find these results.^13,21,26,27^

Most studies investigating cognitive decline in presymptomatic genetic FTD have had a small sample size, a limited number of yearly follow-ups, or did not include all 3 major causes of genetic FTD. Furthermore, most studies split their sample of pathogenic variant carriers either according to the artificial boundary of presymptomatic vs symptomatic or according to estimated years to symptomatic onset. As a result, none of the studies fully highlights the complexity of the disease trajectory.^28^

Larger international cohort studies with longer follow-up time are crucial to identify cognitive markers that signify disease onset at the earliest stage and can measure changes during disease progression. In addition, clinical instruments for disease severity, such as the Clinical Dementia Rating scale plus National Alzheimer's Coordinating Center frontotemporal lobar degeneration module,^29^ could stratify pathogenic variant carriers and provide valuable insight into cognitive decline during the different stages of the disease per genetic group.

This study aims to investigate longitudinal cognitive decline in genetic FTD pathogenic variant carriers. We performed a 5-year follow-up study in which we investigated baseline and longitudinal differences on neuropsychological test performance between *C9orf72*, *GRN*, and *MAPT* pathogenic variant carriers and control participants and stratified pathogenic variant carriers according to the CDR NACC FTLD global score.

## Methods

### Participants

Data were included from the fifth Genetic FTD Initiative (GENFI) data freeze, in which participants from confirmed genetic FTD families were recruited in 24 centers across Europe and Canada between January 30, 2012, and May 31, 2019. Pathogenic variant carriers were included in this study if they performed at least 1 or more neuropsychological assessments. A total of 207 *C9orf72*, 206 *GRN*, and 86 *MAPT* pathogenic variant carriers and 255 pathogenic variant negative family members (who served as control group) were included. A total of 109 *C9orf72*, 112 *GRN*, and 60 *MAPT* pathogenic variant carriers and 154 controls had completed at least 1 follow-up visit (Table 1). Pathogenic variant carriers were divided into 3 categories based on the CDR plus NACC FTLD global score at baseline: 0, 0.5, or ≥1. Of those with a CDR plus NACC FTLD global score of ≥1, 51 *C9orf72*, 27 *GRN*, and 21 *MAPT* pathogenic variant carriers met diagnostic criteria for bvFTD,^2^ 16 *GRN* and 3 *C9orf72* pathogenic variant carriers met criteria for PPA,^3^ and 8 *C9orf72* pathogenic variant carriers met criteria for FTD with amyotrophic lateral sclerosis.^30^ Ten percent of *C9orf72*, 8% of *GRN*, and 8% of *MAPT* pathogenic variant carriers progressed from CDR category 0 to 0.5, and 4% of *C9orf72*, 2% of *GRN*, and 4% of *MAPT* pathogenic variant carriers progressed to ≥1. Six percent of *C9orf72*, 16% of *GRN*, and 20% of *MAPT* pathogenic variant carriers progressed from CDR category 0.5 to ≥1 (eTable 1, links.lww.com/WNL/B987).

**Table 1:**
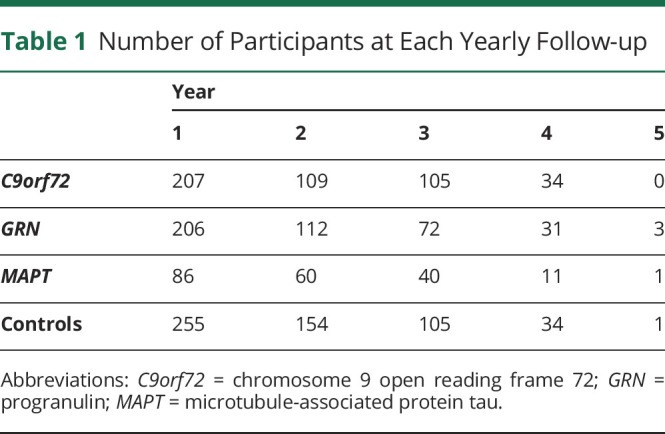
Number of Participants at Each Yearly Follow-up

### Standard Protocol Approvals, Registrations, and Patient Consents

All GENFI sites had local ethical approval for the study and all participants gave written informed consent.

### Procedures

Participants underwent a yearly standardized clinical assessment including the CDR plus NACC FTLD and a comprehensive neuropsychological test battery covering attention and processing speed (Wechsler Memory Scale–Revised [WMS-R] digit span forward^31^; Trail-Making Test [TMT] part A^32^; Wechsler Adult Intelligence Scale–Revised Digit Symbol test^31^; Delis-Kaplan Executive Function System [D-KEFS] Color-Word Interference Test color and word naming^33^), executive function (WMS-R Digit span backward^31^; TMT part B^32^; D-KEFS Color-Word Interference Test ink naming^33^), language (modified Camel and Cactus Test^23^; Boston Naming Test [short 30-item version]^31^), verbal fluency (category fluency^31^; phonemic fluency^34^), memory encoding (Free and Cued Selective Reminding Test [FCSRT] immediate free and total recall^26^), memory recall (FCSRT delayed free and total recall; Benson Complex Figure recall), social cognition (Facial Emotion Recognition Test^24^), and visuoconstruction (Benson Complex Figure copy). Previous studies have shown that verbal fluency can involve both language and executive function processes and therefore we included it as a separate domain.^35,36^ Mini-Mental State Examination (MMSE)^37^ measured global cognitive functioning.

### Statistical Analysis

Statistical analyses were performed using Stata version 14.2 and R version 4.0.4. We compared continuous demographic data between groups with 2-way analyses of variance and a χ^2^ test for sex. The significance level was set at *p* < 0.05 (2-tailed) across all comparisons.

All neuropsychological data were standardized to *z* scores (i.e., raw score − mean score controls at baseline/SD controls at baseline). *z* scores for tests with reaction times (i.e., TMT and D-KEFS Color-Word Interference Test) were inversed so that lower *z* scores indicate worse performance. Cognitive domains were calculated by averaging the mean *z* scores of the neuropsychological tests in that domain. Only the FCSRT total recall was included in the memory domains, as the free recall scores are a part of the total recall scores. The memory, social cognition, and visuoconstruction domains are represented by only 1 test.

As this is a prospective cohort study, not all pathogenic variant carriers had completed all study visits, which resulted in missing data. We used linear mixed-effects models for each cognitive domain to examine whether differences existed between *C9orf72*, *GRN*, and *MAPT* pathogenic variant carriers and controls in cognitive decline since baseline. This type of model allows for the analysis of longitudinal data with unbalanced time points and missing data.^38^ Age and years of education were included in all models as covariates. In each model, a different cognitive outcome was used as the dependent variable and we specified the following fixed effects: time since baseline in years, gene group, CDR category at baseline, age at baseline, years of education, the 2-way interactions between time and group, time and CDR category, and gene group and CDR category, and the 3-way interaction between time, group, and CDR category. We included random intercepts for participants who were nested within families, but not random slopes as this did not improve model fit. A natural cubic splines model did not improve model fit. We performed post hoc pairwise comparisons in intercepts and slopes between genetic groups within CDR categories. Results are shown as a difference between pathogenic variant group and the control group or a different pathogenic variant group if stated. β indicates an estimated difference in *z* score at baseline; β_1_ indicates a difference in change over time (slope). An example of the model and its outputs is shown in eAppendix 1 (links.lww.com/WNL/B987).

### Data Availability

Anonymized data not published within this article will be made available upon reasonable request from any qualified investigator.

## Results

### Demographics

There were more female participants in CDR categories 0 and 0.5 and more male participants in CDR category ≥1 for *C9orf72* (χ2[2] = 9.8, *p* = 0.007) and *MAPT* (χ^2^[2] = 6.6, *p* = 0.036) pathogenic variant carriers (Table 2). We found differences in age at baseline between gene groups (*F*_*3,744*_ = 5.6, *p* < 0.001) and between CDR categories (*F*_*2,744*_ = 91.4, *p* < 0.001) (Table 2). Post hoc pairwise comparisons revealed that *C9orf72* and *GRN* pathogenic variant carriers were older than *MAPT* pathogenic variant carriers (all *p* ≤ 0.02) and controls (all *p* < 0.001) and each CDR category represented older pathogenic variant carriers than the categories with a lower CDR category (all *p* ≤ 0.008). We found differences between CDR categories in years of education at baseline (*F*_*2,744*_ = 8.8, *p* < 0.001), with CDR category ≥1 having had fewer years of education than the other categories (all *p* < 0.03) (Table 2). There was an interaction effect between gene group and CDR category on MMSE at baseline (*F*_*4,742*_ = 4.3, *p* = 0.002). Post hoc simple main effects illustrated a difference in MMSE at baseline between CDR categories in all 3 gene groups and a difference in MMSE at baseline between gene groups in CDR category ≥1. Descriptive and neuropsychological data at baseline are reported in Table 2 and eTable 2 (links.lww.com/WNL/B987).

**Table 2:**
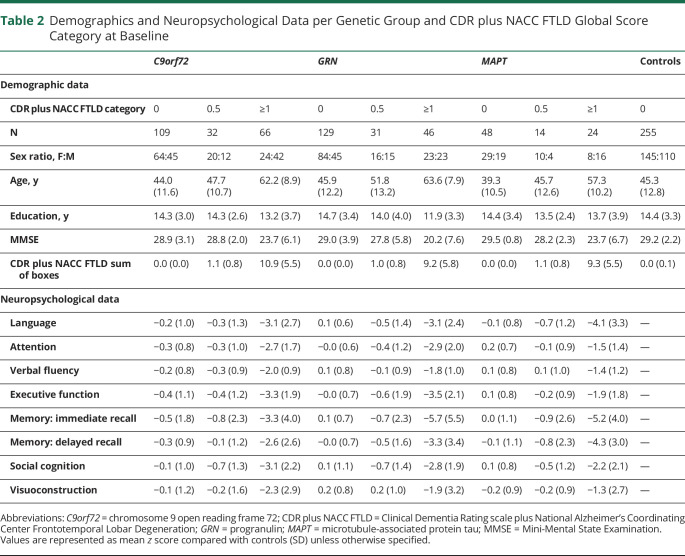
Demographics and Neuropsychological Data per Genetic Group and CDR plus NACC FTLD Global Score Category at Baseline

**Figure 1:**
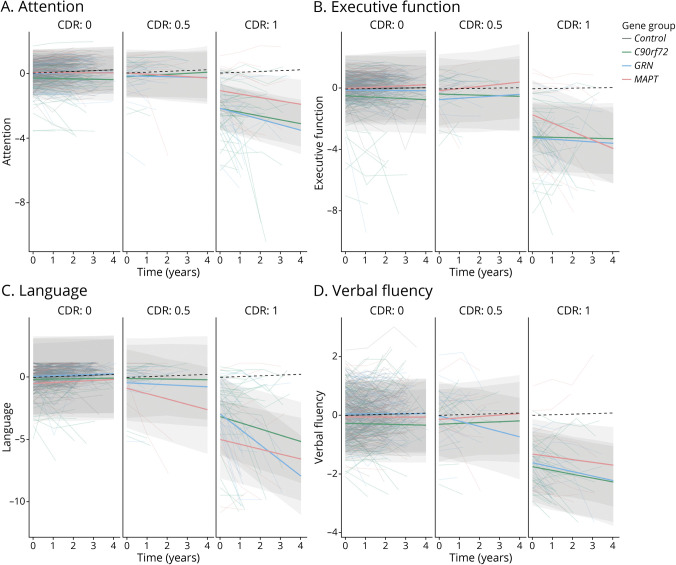
Linear Mixed Effects Models Displaying Longitudinal Trajectories in Composite Domain *Z* Score Stratified by the CDR Plus NACC FTLD for *C9orf72*, *GRN*, and *MAPT* Pathogenic Variant Carriers and Healthy Controls Models are displayed per cognitive domain: (A) attention, (B) executive function, (C) language, and (D) verbal fluency. *C9orf72* = chromosome 9 open reading frame 72; CDR = Clinical Dementia Rating scale plus National Alzheimer's Coordinating Center Frontotemporal Lobar Degeneration; *GRN* = progranulin; *MAPT* = microtubule-associated protein tau.

**Figure 2:**
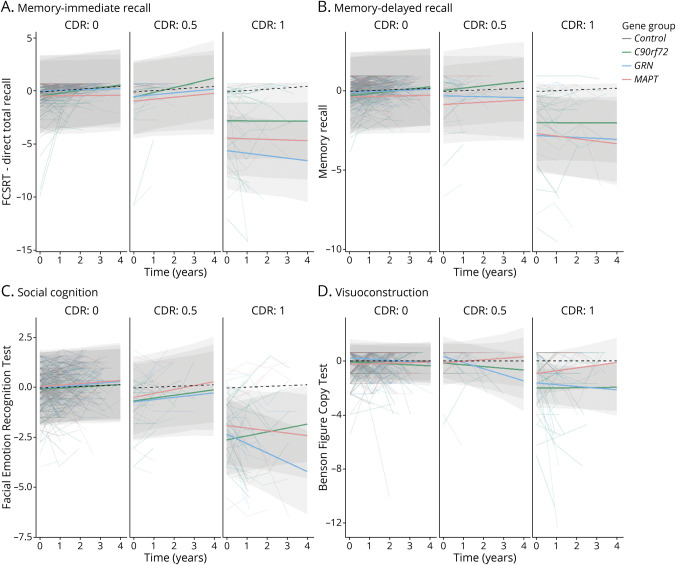
Linear Mixed Effects Models Displaying Longitudinal Trajectories in Composite Domain *Z* Score Stratified by the CDR Plus NACC FTLD for *C9orf72*, *GRN*, and *MAPT* Pathogenic Variant Carriers and Healthy Controls Models are displayed per cognitive domain: (A) memory–immediate recall, (B) memory–delayed recall, (C) social cognition, and (D) visuoconstruction. *C9orf72* = chromosome 9 open reading frame 72; CDR = Clinical Dementia Rating scale plus National Alzheimer's Coordinating Center Frontotemporal Lobar Degeneration; *GRN* = progranulin; *MAPT* = microtubule-associated protein tau.

**Figure 3:**
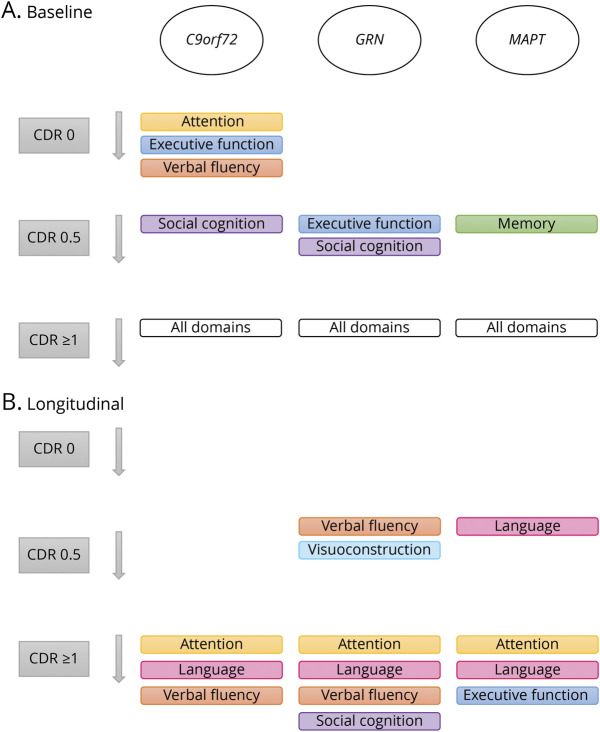
Summary of Cross-Sectional and Longitudinal Differences Between Each Genetic Group and Controls (A) Cross-sectional differences. (B) Longitudinal differences. *C9orf72* = chromosome 9 open reading frame 72; CDR = Clinical Dementia Rating scale plus National Alzheimer's Coordinating Center Frontotemporal Lobar Degeneration; *GRN* = progranulin; *MAPT* = microtubule-associated protein tau.

Baseline and longitudinal results for each cognitive domain are discussed in the following sections (Tables 2 and 3, Figures 1 and 2, and summarized in Figure 3).

**Table 3:**
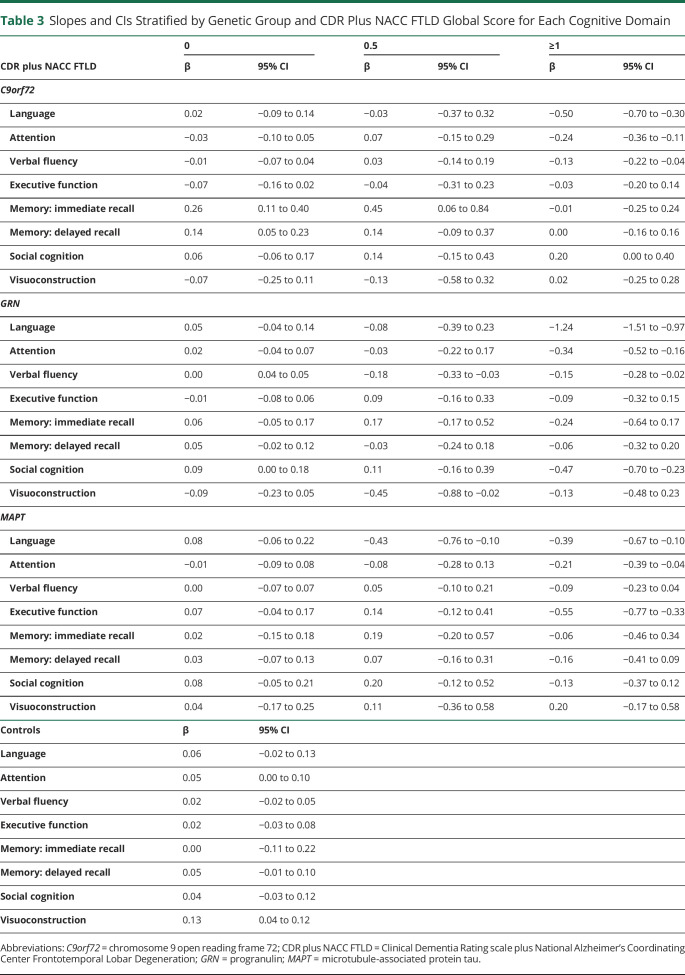
Slopes and CIs Stratified by Genetic Group and CDR Plus NACC FTLD Global Score for Each Cognitive Domain

### Attention

We found strong evidence for differences in the attention domain between CDR categories (χ2[2] = 23.2, *p* < 0.001) and between gene groups (χ^2^[3] = 26.0, *p* < 0.001) at baseline. *C9orf72* (β = −2.2, SE 0.14, *p* < 0.001), *GRN* (β = −2.2, SE 0.16, *p* < 0.001), and *MAPT* (β = −1.1, SE 0.21, *p* < 0.001) pathogenic variant carriers with CDR category ≥1 all performed worse than controls, with both *C9orf72* (β = −1.1, SE 0.23, *p* < 0.001) and *GRN* (β = −1.2, SE 0.25, *p* < 0.001) pathogenic variant carriers performing worse than *MAPT* pathogenic variant carriers. *C9orf72* pathogenic variant carriers with CDR category 0 also performed worse at baseline than *GRN* (β = −0.3, SE 0.13, *p* = 0.010) and *MAPT* (β = −0.4, SE 0.16, *p* = 0.030) pathogenic variant carriers and controls (β = −0.4, SE 0.11, *p* < 0.001; Figure 1A). In addition, we found an interaction effect between time and gene group (χ^2^[3] = 37.1, *p* < 0.001). All gene groups with CDR category ≥1 declined over time compared with controls (*C9orf72*: β_1_ = −0.3, SE 0.07, *p* < 0.001; *GRN*: β_1_ = −0.4, SE 0.10, *p* < 0.001; *MAPT*: β_1_ = −0.3, SE 0.09, *p* = 0.004). There was some weak evidence that *C9orf72* pathogenic variant carriers with CDR category 0 declined over time compared with controls (β_1_ = −0.4, SE 0.11, *p* = 0.086; Figure 1A).

### Executive Function

We found strong evidence for differences on the executive function domain between CDR categories (χ^2^[2] = 27.2, *p* < 0.001) and between gene groups (χ^2^[3] = 23.3, *p* < 0.001) at baseline. A similar profile was seen in all gene groups with CDR category ≥1 performing worse at baseline than controls (*C9orf72*: β = −3.1, SE 0.25, *p* < 0.001; *GRN*: β = −3.2, SE 0.23, *p* < 0.001; *MAPT*: β = −1.7, SE 0.29, *p* < 0.001), and *C9orf72* (β = −1.0, SE 0.32, *p* = 0.003) and *GRN* (β = −1.1, SE 0.35, *p* = 0.002) pathogenic variant carriers performing worse than *MAPT* pathogenic variant carriers (Figure 1B). *C9orf72* pathogenic variant carriers with CDR category 0 also performed worse than *GRN* (β = −0.4, SE 0.17, *p* = 0.016) and *MAPT* (β = −0.6, SE 0.23, *p* = 0.012) pathogenic variant carriers, and controls (β = −0.5, SE 0.15, *p* < 0.001) and *GRN* pathogenic variant carriers with CDR category 0.5 performed worse than controls (β = −0.7, SE 0.25, *p* = 0.006). We found interaction effects between time and gene group (χ^2^[3] = 24.7, *p* < 0.001), time and CDR category (χ^2^[2] = 25.8, *p* < 0.001), and time, gene group, and CDR category (χ^2^[4] = 18.6, *p* = 0.001). *MAPT* pathogenic variant carriers with CDR category ≥1 demonstrated steeper decline over time than *C9orf72* (β_1_ = −0.5, SE 0.14, *p* = 0.002) and *GRN* pathogenic variant carriers (β_1_ = −0.5, SE 0.17, *p* = 0.005) and controls (β_1_ = −0.6, SE 0.12, *p* < 0.001) (Figure 1B).

### Language

Language differed between CDR categories (χ2[2] = 96.7, *p* < 0.001) and between gene groups (χ^2^[3] = 21.5, *p* < 0.001) at baseline. Again, all gene groups with CDR category ≥1 performed worse than controls (*C9orf72*: β = −3.2, SE 0.28, *p* < 0.001; *GRN*: β = −2.9, SE 0.31, *p* < 0.001; *MAPT*: β = −5.0, SE 0.41, *p* < 0.001) at baseline, but in this case *MAPT* pathogenic variant carriers performed worse than *C9orf72* (β = −1.7, SE 0.34, *p* = 0.002) and *GRN* (β = −1.3, SE 0.33, *p* = 0.009) pathogenic variant carriers (Figure 1C). We also found interaction effects between time and gene group (χ^2^[3] = 104.8, *p* < 0.001), time and CDR category (χ^2^[2] = 14.0, *p* = 0.001), and time, gene group, and CDR category (χ^2^[4] = 25.5, *p* < 0.001). *MAPT* pathogenic variant carriers with CDR category 0.5 (β_1_ = −0.5, SE 0.17, *p* = 004) or ≥1 (β_1_ = −0.5, SE 0.15, *p* = 0.003) as well as *C9orf72* (β_1_ = −0.6, SE 0.11, *p* < 0.001) and *GRN* (β_1_ = −1.3, SE 0.14, *p* < 0.001) pathogenic variant carriers with CDR category ≥1 declined over time compared with controls. In CDR category ≥1, *GRN* pathogenic variant carriers demonstrated steeper decline over time than *C9orf72* (β_1_ = −0.7, SE 0.17, *p* < 0.001) and *MAPT* (β_1_ = −0.9, SE 0.20, *p* < 0.001) pathogenic variant carriers (Figure 1C).

### Verbal Fluency

For verbal fluency, we found strong evidence for differences between CDR categories (χ2[2] = 40.0, *p* < 0.001) at baseline. All gene groups with CDR category ≥1 performed worse than controls (*C9orf72*: β = −1.8, SE 0.12, *p* < 0.001; *GRN*: β = −1.6, SE 0.14, *p* < 0.001; *MAPT*: β = −1.3, SE 0.18, *p* < 0.001), with *C9orf72* performing worse than *MAPT* pathogenic variant carriers (β = −0.5, SE 0.19, *p* = 0.018; Figure 1D). In CDR category 0, *C9orf72* pathogenic variant carriers performed worse than controls (β = −0.3, SE 0.09, *p* = 0.003) and *GRN* pathogenic variant carriers (β = −0.3, SE 0.11, *p* = 0.002). We found an interaction effect between time and gene group (χ^2^[3] = 14.5, *p* < 0.002). *C9orf72* pathogenic variant carriers with CDR category ≥1 (β_1_ = −0.2, SE 0.05, *p* = 0.004) and *GRN* pathogenic variant carriers with CDR categories 0.5 (β_1_ = −0.2, SE 0.08, *p* = 0.013) and ≥1 (β_1_ = −0.2, SE 0.07, *p* = 0.015) declined over time compared with controls (Figure 1D).

### Memory: Immediate Recall

For immediate recall, we found strong evidence for differences between CDR categories (χ^2^[2] = 51.4, *p* < 0.001) and between gene groups (χ^2^[3] = 40.2, *p* < 0.001) at baseline. All gene groups with CDR category ≥1 performed worse than controls (*C9orf72*: β = −2.7, SE 0.32, *p* < 0.001; *GRN*: β = −5.5, SE 0.40, *p* < 0.001; *MAPT*: β = −4.3, SE 0.51, *p* < 0.001), with *MAPT* performing worse than *C9orf72* pathogenic variant carriers (β = −1.7, SE 0.56, *p* = 0.003) and *GRN* pathogenic variant carriers performing worse than *C9orf72* (β = −3.0, SE 0.47, *p* < 0.001) and *MAPT* pathogenic variant carriers (β = −1.2, SE 0.62, *p* = 0.032; Figure 2A).

### Memory: Delayed Recall

For delayed recall, we also found evidence for differences between CDR categories (χ2[2] = 36.9, *p* < 0.001) and between gene groups (χ^2^[3] = 10.4, *p* = 0.015) at baseline. Again, all gene groups with CDR category ≥1 performed worse than controls (*C9orf72*: β = −2.0, SE 0.21, *p* < 0.001; *GRN*: β = −2.8, SE 0.27, *p* < 0.001; *MAPT*: β = −2.7, SE 0.35, *p* < 0.001), with *GRN* (β = −0.9, SE 0.32, *p* = 0.007) and *MAPT* (β = −0.8, SE 0.38, *p* = 0.033) performing worse than *C9orf72* pathogenic variant carriers. *MAPT* pathogenic variant carriers with CDR category 0.5 (β = −0.8, SE 0.36, *p* = 0.021) performed worse than controls and *C9orf72* pathogenic variant carriers (β = −0.9, SE 0.42, *p* = 0.023). There was some weak evidence indicating that *MAPT* pathogenic variant carriers with CDR category 0 performed worse than controls (β = −0.4, SE 0.21, *p* = 0.081; Figure 2B). None of the groups declined significantly over time.

### Social Cognition

We found strong evidence for differences between CDR categories (χ^2^[2] = 35.7, *p* < 0.001) at baseline on social cognition. All gene groups with CDR category ≥1 performed worse than controls (*C9orf72*: β = −2.6, SE 0.19, *p* < 0.001; *GRN*: β = −2.3, SE 0.23, *p* < 0.001; *MAPT*: β = −1.9, SE 0.28, *p* < 0.001), with *GRN* performing worse than *MAPT* pathogenic variant carriers (β = −0.7, SE 0.33, *p* = 0.033; Figure 2C). *C9orf72* (β = −0.7, SE 0.24, *p* = 0.001) and *GRN* (β = −0.7, SE 0.25, *p* = 0.001) pathogenic variant carriers with CDR category 0.5 also performed worse at baseline than controls. We found interaction effects between time and gene group (χ^2^[3] = 21.3, *p* < 0.001) and time, CDR category, and gene group (χ^2^[4] = 16.3, *p* < 0.003). *GRN* pathogenic variant carriers with CDR category ≥1 showed steeper decline over time compared with controls (β_1_ = −0.5, SE 0.13, *p* < 0.001), *C9orf72* (β_1_ = −0.7, SE 0.16, *p* < 0.001), and *MAPT* (β_1_ = −0.3, SE 0.17, *p* = 0.049) pathogenic variant carriers and *MAPT* pathogenic variant carriers with CDR category ≥1 showed steeper decline over time compared to *C9orf72* pathogenic variant carriers (β_1_ = −0.3, SE 0.16, *p* = 0.047; Figure 2C).

### Visuoconstruction

We found differences between gene groups on visuoconstruction (χ^2^[3] = 11.0, *p* = 0.012) at baseline. All gene groups with CDR category ≥1 performed worse than controls (*C9orf72*: β = −2.0, SE 0.22, *p* < 0.001; *GRN*: β = −1.6, SE 0.26, *p* < 0.001; *MAPT*: β = −0.9, SE 0.32, *p* = 0.004), with *C9orf72* (β = −1.2, SE 0.33, *p* = 0.002) and *GRN* (β = −1.0, SE 0.36, *p* = 0.008) performing worse than *MAPT* pathogenic variant carriers. *GRN* pathogenic variant carriers with CDR category 0.5 (β_1_ = −0.5, SE 0.23, *p* = 0.050) showed steeper decline over time than controls (Figure 2D).

## Discussion

This study demonstrated gene-specific baseline differences and decline over a 5-year time period in a large cohort of genetic FTD pathogenic variant carriers that was moderated by the CDR plus NACC FTLD global score. *C9orf72* pathogenic variant carriers performed lower on attention, executive function, and verbal fluency from CDR plus NACC FTLD 0 onwards, with relatively minimal decline over time compared with other genetic groups regardless of the CDR plus NACC FTLD score (i.e., disease progression). The cognitive profile in *MAPT* pathogenic variant carriers was characterized by early impaired memory (already at CDR plus NACC FTLD 0.5), with language decline starting at CDR plus NACC FTLD 0.5, and executive dysfunction developing rapidly at CDR plus NACC FTLD ≥1. *GRN* pathogenic variant carriers showed no differences or decline compared with controls at CDR plus NACC FTLD 0, but verbal fluency and visuoconstruction started to decline at CDR plus NACC FTLD 0.5. *GRN* pathogenic variant carriers showed the most rapid decline compared with the other groups in language and social cognition from CDR plus NACC FTLD ≥1 onwards. The results from this study confirm cognitive decline in the asymptomatic and prodromal stages of genetic FTD and hold potential for upcoming therapeutic trials by identifying (1) the most sensitive cognitive measures to track disease progression and treatment effects and (2) the speed of change over time, thereby providing insight into the best time window to start disease-modifying treatment.

Asymptomatic *C9orf72* pathogenic variant carriers performed worse at baseline than controls on attention/mental processing speed, executive function, and verbal fluency. In the prodromal stage, social cognition was also lower at baseline, whereas at the fully symptomatic stage, all cognitive domains were lower at baseline. There was no decline over time in the asymptomatic stage or prodromal stage, but attention/mental processing speed, language, and verbal fluency declined over time in the symptomatic stage, although less rapidly than in other gene groups. The other cognitive domains remained relatively stable, and there were signs of possible practice effects for memory and social cognition. This is largely in line with previous studies demonstrating widespread cognitive impairment in *C9orf72* pathogenic variant carriers with relatively minimal decline over time.^5,39,40^ It is further corroborated by the fact that the neurodegenerative process associated with the *C9orf72* pathogenic variant is widespread, with neurodegeneration in the frontal and temporal cortices but also in more posterior cortical, subcortical, and cerebellar regions.^39,41^ This group performed lowest compared with the other groups on a wide range of neuropsychological tests, specifically tests for attention/mental processing speed and executive function, at the asymptomatic stage. Although these performances were not at an “impaired” level (i.e., *z* score ≤−2), these deficits might represent the earliest signs of neurodegeneration with very slow decline over time. Alternatively, the lack of decline over time in all 3 disease stages raises the intriguing possibility that these deficits are not merely preclinical signs of FTD as a result of early neurodegeneration, but might be indicative of a neurodevelopmental disorder in *C9orf72*, which at a certain age is superimposed by additional neurodegeneration. This hypothesis has been suggested by several previous studies that found gray and white matter deficits and connectivity disruption as well as psychiatric conditions and cognitive deficits many years before the estimated age at symptom onset without evidence of disease progression over time.^42,43^ Future studies should focus on ascertaining early-life radiologic and clinical assessments to test this hypothesis.

In *MAPT* pathogenic variant carriers, there was a trend towards lower memory performance than in controls at baseline in the asymptomatic stage, which became significant at the prodromal stage. All cognitive domains were lower than in controls at baseline in the symptomatic stage. There was no decline over time in the asymptomatic stage, but language declined from the prodromal stage onwards. In addition, attention/mental processing speed, executive function, and social cognition declined progressively during the symptomatic stage. These results confirm that the first changes for this group occur in cognitive functions that are strongly associated with the temporal lobe, an area that already shows early degeneration in presymptomatic *MAPT* pathogenic variant carriers.^6^ Several previous studies have demonstrated that episodic memory impairment is a distinct feature in *MAPT*-related FTD, even in presymptomatic pathogenic variant carriers.^19,20,26^ Strikingly, we demonstrated lower memory performance in prodromal pathogenic variant carriers but with practice effects over time that disappeared at the fully symptomatic stage only. A likely explanation for these practice effects is that the same items for memory tests were used at all time points, stressing the need for the use of tests that have multiple versions with different stimuli in longitudinal cohort studies. The lower performance and decline seen in the language domain was largely driven by the Boston Naming Test, a test that strongly depends on the semantic memory system.^44^ This is unsurprising given that semantic memory is strongly associated with the anteromedial temporal lobe, an area known to deteriorate early and progressively in *MAPT*-associated FTD.^26^ Deficits in semantic memory have been described as a key symptom in *MAPT* pathogenic variant carriers in a more progressed disease stage,^5^ but our results illustrate that the first changes occur at a much earlier stage, suggesting that semantic tests might be a good candidate to serve as a sensitive end point in upcoming therapeutic trials of *MAPT*-associated FTD. Only at a later progressed stage, when atrophy spreads from the temporal to frontal areas of the brain, does impairment in cognitive functions that are typically associated with bvFTD develop, such as executive function and social cognition.^22,45^

There were no cross-sectional differences between asymptomatic *GRN* pathogenic variant carriers and controls at baseline and there was no decline over time in this stage. In the prodromal stage, pathogenic variant carriers performed worse than controls on executive function and social cognition, and they declined over time on verbal fluency and visuoconstruction. All cognitive domains were lower than in controls at baseline in the symptomatic stage, and they showed progressive decline over time on attention/mental processing speed, verbal fluency, language, and social cognition. This is in line with previous studies showing minimal changes in gray and white matter but also cognition in presymptomatic *GRN* pathogenic variant carriers, often with fast progressive decline after symptom onset.^5,20^ Although in our study no change over time was detected in the asymptomatic stage, *GRN* pathogenic variant carriers performed worse on executive function and social cognitive tasks at the prodromal stage, suggesting some decline between these stages. Possible explanations could be that the asymptomatic pathogenic variant carriers were too far from symptom onset or that the time window between these stages where these changes occur is relatively short. Interestingly, verbal fluency declined progressively in the prodromal period, indicating an early deficit in specifically verbal fluency. This could be interpreted as an early sign of pathogenic variant carriers developing nonfluent variant PPA, a clinical phenotype that is often seen in *GRN* pathogenic variant carriers.^41^ However, verbal fluency measures are also known to strongly depend on executive function,^36^ a cognitive domain known to deteriorate in bvFTD.^45^ Surprisingly, visuoconstruction also declined in the prodromal stage, whereas this is considered to be relatively spared in FTD.^2^ However, most visuoconstructive tasks also strongly depend on executive functions such as planning, organizing, and keeping overview.^46^ It seems, therefore, more likely that these tasks were influenced by impaired executive function rather than a pure impairment in language and visuoconstruction per se.

This is the first study to longitudinally investigate a large cohort of all 3 major causes of genetic FTD over a 5-year period. A major strength of this study is the use of the CDR plus NACC FTLD to stratify pathogenic variant carriers from asymptomatic to prodromal and fully symptomatic (i.e., 0, 0.5, ≥1). Most previous studies have stratified pathogenic variant carriers as either presymptomatic or symptomatic according to whether they fulfilled diagnostic criteria for FTD syndromes, but this does not fully grasp the clinical trajectory of FTD. The cognitive profile between the presymptomatic and symptomatic phase has not been well-characterized. Some other studies have used estimated years to symptom onset based on mean family age at onset, but a recent article demonstrated that the correlations between age at symptom onset and mean family age at symptom onset were weak for *C9orf72* and *GRN* pathogenic variant carriers, indicating that this might not be a reliable proxy.^28^ By stratifying according to CDR plus NACC FTLD, we have provided insight into cognitive decline during different disease stages.

There are limitations to this study. First, the sample size at the CDR plus NACC FTLD 0.5 stage was smaller than the other stages, which probably influenced the statistical power in this specific group. Second, due to ongoing recruitment within GENFI, participants varied in the number of completed visits, resulting in missing data at later time points. Therefore, we analyzed the data with linear mixed-effects models, as these models allow for unbalanced time points and missing data.^38^ We could not use a nonlinear mixed effects model (e.g., natural cubic splines) due to the limited number of follow-up visits. However, similar to what has been performed in studies of familial AD,^47^ nonlinear models might be more suitable for the analysis of clinical progression in FTD. Future studies with longer follow-up should therefore investigate the use of nonlinear models in analyzing clinical disease progression in FTD. Third, we did not take progression over time on the CDR plus NACC FTLD into account, but stratified groups according to their global score at baseline. Future research should investigate the cognitive trajectories of progressors compared with nonprogressors on the CDR plus NACC FTLD more in depth. Individual trajectories demonstrated high variability between individuals in each group. A possible explanation for this interindividual variability could be that some individuals with a CDR plus NACC FTLD global score of 0 might be closer to symptom onset than others. Similarly, individuals with a CDR plus NACC FTLD score of 0.5 or ≥1 at baseline might vary in time since progression to that CDR category (i.e., individuals who had a global score of 0.5 for several years at inclusion will likely progress faster than individuals who progressed to a score of 0.5 more recently). Validation in other cohorts such as ALLFTD or DINAD is warranted. Fourth, practice effects were strikingly visible for the FCSRT and Facial Emotion Recognition Test, stressing the need for different test versions in the former, but more sensitive tasks for emotion recognition (e.g., the use of morphed facial expressions^22^) and social cognition in general. Lastly, in the interpretation of memory–immediate recall, social cognition, and visuoconstruction results, it should be taken into account that they were represented by only a single cognitive test, and those individual tests might not be a representation of the entire cognitive domain.

We provide evidence for gene-specific cognitive decline in the prodromal stage of genetic FTD. Specifically, tests for attention/mental processing speed, executive function, language, and memory showed clear differences between gene groups and controls at baseline, but the speed and nature of change over time differed depending on (1) the gene group and (2) the CDR plus NACC FTLD global score. These results confirm the value of neuropsychological assessment in tracking disease progression and could inform upcoming clinical trials in selecting sensitive end points for measuring treatment effects as well as in characterizing the best time window for starting treatment.

## Glossary

bvFTD: behavioral variant frontotemporal dementia
*C9orf72*: chromosome 9 open reading frame 72
CDR plus NACC FTLD: Clinical Dementia Rating (CDR) scale plus National Alzheimer's Coordinating Center (NACC) frontotemporal lobar degeneration
D-KEFS: Delis-Kaplan Executive Function System
FCSRT: Free and Cued Selective Reminding Test
FTD: frontotemporal dementia
GENFI: Genetic FTD Initiative
*GRN*: progranulin
*MAPT*: microtubule-associated protein tau
MMSE: Mini-Mental State Examination
PPA: primary progressive aphasia
TMT: Trail-Making Test
WMS-R: Wechsler Memory Scale–Revised

## References

[1] Seelaar H, Rohrer JD, Pijnenburg YA, Fox NC, van Swieten JC. Clinical, genetic and pathological heterogeneity of frontotemporal dementia: a review. J Neurol Neurosurg Psychiatry. 2011;82(5):476-486. 20971753 10.1136/jnnp.2010.212225

[2] Rascovsky K, Hodges JR, Knopman D, et al. Sensitivity of revised diagnostic criteria for the behavioural variant of frontotemporal dementia. Brain. 2011;134(pt 9):2456-2477. 21810890 10.1093/brain/awr179 PMC3170532

[3] Gorno-Tempini ML, Hillis AE, Weintraub S, et al. Classification of primary progressive aphasia and its variants. Neurology. 2011;76(11):1006-1014. 21325651 10.1212/WNL.0b013e31821103e6 PMC3059138

[4] Lashley T, Rohrer JD, Mead S, Revesz T. An update on clinical, genetic and pathological aspects of frontotemporal lobar degenerations. Neuropathol Appl Neurobiol. 2015;41(7):858-881. 26041104 10.1111/nan.12250

[5] Poos JM, Jiskoot LC, Leijdesdorff SMJ, et al. Cognitive profiles discriminate between genetic variants of behavioral frontotemporal dementia. J Neurol. 2020;267(6):1603-1612. 32052166 10.1007/s00415-020-09738-y PMC7293665

[6] Rohrer JD, Nicholas JM, Cash DM, et al. Presymptomatic cognitive and neuroanatomical changes in genetic frontotemporal dementia in the Genetic Frontotemporal Dementia Initiative (GENFI) study: a cross-sectional analysis. Lancet Neurol. 2015;14(3):253-262. 25662776 10.1016/S1474-4422(14)70324-2 PMC6742501

[7] Jiskoot LC, Panman JL, Meeter LH, et al. Longitudinal multimodal MRI as prognostic and diagnostic biomarker in presymptomatic familial frontotemporal dementia. Brain. 2018;142(1):193-208. 10.1093/brain/awy288 PMC6308313 30508042

[8] van der Ende EL, Meeter LH, Poos JM, et al. Serum neurofilament light chain in genetic frontotemporal dementia: a longitudinal, multicentre cohort study. Lancet Neurol. 2019;18(12):1103-1111. 31701893 10.1016/S1474-4422(19)30354-0

[9] Panman JL, Jiskoot LC, Bouts M, et al. Gray and white matter changes in presymptomatic genetic frontotemporal dementia: a longitudinal MRI study. Neurobiol Aging. 2019;76:115-124. 30711674 10.1016/j.neurobiolaging.2018.12.017

[10] Benussi A, Gazzina S, Premi E, et al. Clinical and biomarker changes in presymptomatic genetic frontotemporal dementia. Neurobiol Aging. 2019;76:133-140. 30711676 10.1016/j.neurobiolaging.2018.12.018

[11] Mutsaerts HJMM, Mirza SS, Petr J, et al. Cerebral perfusion changes in presymptomatic genetic frontotemporal dementia: a GENFI study. Brain. 2019;142(4):1108-1120. 30847466 10.1093/brain/awz039 PMC6439322

[12] Cash DM, Bocchetta M, Thomas DL, et al. Patterns of gray matter atrophy in genetic frontotemporal dementia: results from the GENFI study. Neurobiol Aging. 2018;62:191-196. 29172163 10.1016/j.neurobiolaging.2017.10.008 PMC5759893

[13] Dopper EGP, Rombouts SARB, Jiskoot LC, et al. Structural and functional brain connectivity in presymptomatic familial frontotemporal dementia. Neurology. 2014;83(2):e19-e26. 25002573 10.1212/WNL.0000000000000583

[14] Tsai RM, Boxer AL. Therapy and clinical trials in frontotemporal dementia: past, present, and future. J Neurochem. 2016;138(suppl 1):211-221. 27306957 10.1111/jnc.13640 PMC5217534

[15] Barandiaran M, Estanga A, Moreno F, et al. Neuropsychological features of asymptomatic c. 709-1G> A progranulin mutation carriers. J Int Neuropsychol Soc. 2012;18(6):1086-1090. 23158232 10.1017/S1355617712000823

[16] Barandiaran M, Moreno F, de Arriba M, et al. Longitudinal neuropsychological study of presymptomatic c. 709-1G> A progranulin mutation carriers. J Int Neuropsychol Soc. 2019;25(1):39-47. 30369339 10.1017/S1355617718000735

[17] Cheran G, Wu L, Lee S, et al. Cognitive indicators of preclinical behavioral variant frontotemporal dementia in MAPT carriers. J Int Neuropsychol Soc. 2019;25(2):184-194. 30458895 10.1017/S1355617718001005 PMC6374161

[18] Hallam BJ, Jacova C, Hsiung GYR, et al. Early neuropsychological characteristics of progranulin mutation carriers. J Int Neuropsychol Soc. 2014;20(7):694-703. 24993774 10.1017/S1355617714000551

[19] Jiskoot LC, Dopper EG, Heijer T, et al. Presymptomatic cognitive decline in familial frontotemporal dementia: a longitudinal study. Neurology. 2016;87(4):384-391. 27358337 10.1212/WNL.0000000000002895

[20] Jiskoot LC, Panman JL, van Asseldonk L, et al. Longitudinal cognitive biomarkers predicting symptom onset in presymptomatic frontotemporal dementia. J Neurol. 2018;265(6):1381-1392. 29627938 10.1007/s00415-018-8850-7 PMC5990575

[21] Papma JM, Jiskoot LC, Panman JL, et al. Cognition and gray and white matter characteristics of presymptomatic C9orf72 repeat expansion. Neurology. 2017;89(12):1256-1264. 28855404 10.1212/WNL.0000000000004393

[22] Jiskoot LC, Poos JM, Vollebergh ME, et al. Emotion recognition of morphed facial expressions in presymptomatic and symptomatic frontotemporal dementia, and Alzheimer's dementia. J Neurol. 2021;268(1):102-113. 32728945 10.1007/s00415-020-10096-y PMC7815624

[23] Moore K, Convery R, Bocchetta M, et al. A modified Camel and Cactus Test detects presymptomatic semantic impairment in genetic frontotemporal dementia within the GENFI cohort. Appl Neuropsychol Adult. 2022;29(1):112-119. 32024404 10.1080/23279095.2020.1716357

[24] Russell LL, Greaves CV, Bocchetta M, et al. Social cognition impairment in genetic frontotemporal dementia within the GENFI cohort. Cortex. 2020;133:384-398. 33221702 10.1016/j.cortex.2020.08.023 PMC7754789

[25] Franklin HD, Russell LL, Peakman G, et al. The revised self-monitoring scale detects early impairment of social cognition in genetic frontotemporal dementia within the GENFI cohort. Alzheimers Res Ther. 2021;13(1):127. 34253227 10.1186/s13195-021-00865-w PMC8276486

[26] Poos JM, Russell LL, Peakman G, et al. Impairment of episodic memory in genetic frontotemporal dementia: a GENFI study. Alzheimers Dement. 2021;13(1):e12185. 10.1002/dad2.12185 PMC8116844 34027016

[27] Bertrand A, Wen J, Rinaldi D, et al. Early cognitive, structural, and microstructural changes in presymptomatic C9orf72 carriers younger than 40 years. JAMA Neurol. 2018;75(2):236-245. 29197216 10.1001/jamaneurol.2017.4266 PMC5838615

[28] Moore KM, Nicholas J, Grossman M, et al. Age at symptom onset and death and disease duration in genetic frontotemporal dementia: an international retrospective cohort study. Lancet Neurol. 2020;19(2):145-156. 31810826 10.1016/S1474-4422(19)30394-1 PMC7007771

[29] Miyagawa T, Brushaber D, Syrjanen J, et al. Utility of the global CDR plus NACC FTLD rating and development of scoring rules: data from the ARTFL/LEFFTDS Consortium. Alzheimers Dement. 2020;16(1):106-117. 31914218 10.1002/alz.12033 PMC7202045

[30] Brooks BR, Miller RG, Swash M, Munsat TL; World Federation of Neurology Research Group on Motor Neuron Diseases. El Escorial revisited: revised criteria for the diagnosis of amyotrophic lateral sclerosis. Amyotroph Lateral Scler Other Motor Neuron Disord. 2000;1(5):293-299. 11464847 10.1080/146608200300079536

[31] Morris JC, Weintraub S, Chui HC, et al. The uniform data set (UDS): clinical and cognitive variables and descriptive data from Alzheimer disease centers. Alzheimer Dis Assoc Disord. 2006;20(4):210-216. 17132964 10.1097/01.wad.0000213865.09806.92

[32] Corrigan JD, Hinkeldey NS. Relationships between parts A and B of the Trail Making Test. J Clin Psychol. 1987;43(4):402-409. 3611374 10.1002/1097-4679(198707)43:4<402::aid-jclp2270430411>3.0.co;2-e

[33] Delis DC, Kaplan E, Kramer J, den Buysch HO, Noens ILJ, Berckelaer-Onnes IA. D-KEFS: Delis-Kaplan Executive Function System: Color-Word Interference Test: Handleiding. Pearson; 2008.

[34] Tombaugh TN, Kozak J, Rees L. Normative data stratified by age and education for two measures of verbal fluency: FAS and animal naming. Arch Clin Neuropsychol. 1999;14(2):167-177. 14590600

[35] Shao Z, Janse E, Visser K, Meyer AS. What do verbal fluency tasks measure? Predictors of verbal fluency performance in older adults. Front Psychol. 2014;5:772. 25101034 10.3389/fpsyg.2014.00772 PMC4106453

[36] Whiteside DM, Kealey T, Semla M, et al. Verbal fluency: language or executive function measure? Appl Neuropsychol Adult. 2016;23(1):29-34. 26111011 10.1080/23279095.2015.1004574

[37] Folstein MF, Folstein SE, McHugh PR. “Mini-mental state”: a practical method for grading the cognitive state of patients for the clinician. J Psychiatr Res. 1975;12(3):189-198. 1202204 10.1016/0022-3956(75)90026-6

[38] Cnaan A, Laird NM, Slasor P. Using the general linear mixed model to analyse unbalanced repeated measures and longitudinal data. Stat Med. 1997;16(20):2349-2380. 9351170 10.1002/(sici)1097-0258(19971030)16:20<2349::aid-sim667>3.0.co;2-e

[39] Mahoney CJ, Downey LE, Ridgway GR, et al. Longitudinal neuroimaging and neuropsychological profiles of frontotemporal dementia with C9ORF72 expansions. Alzheimers Res Ther. 2012;4(5):41. 23006986 10.1186/alzrt144 PMC3580398

[40] Khan BK, Yokoyama JS, Takada LT, et al. Atypical, slowly progressive behavioural variant frontotemporal dementia associated with C9ORF72 hexanucleotide expansion. J Neurol Neurosurg Psychiatry. 2012;83(4):358-364. 22399793 10.1136/jnnp-2011-301883 PMC3388906

[41] Rohrer JD, Warren JD. Phenotypic signatures of genetic frontotemporal dementia. Curr Opin Neurol. 2011;24(6):542-549. 21986680 10.1097/WCO.0b013e32834cd442

[42] Lee SE, Sias AC, Mandelli ML, et al. Network degeneration and dysfunction in presymptomatic C9ORF72 expansion carriers. Neuroimage Clin. 2017;14:286-297. 28337409 10.1016/j.nicl.2016.12.006 PMC5349617

[43] Lulé DE, Müller HP, Finsel J, et al. Deficits in verbal fluency in presymptomatic C9orf72 mutation gene carriers: a developmental disorder. J Neurol Neurosurg Psychiatry. 2020;91(11):1195-1200. 32855285 10.1136/jnnp-2020-323671 PMC7569387

[44] Kaplan E, Goodglass H, Weintraub S. Boston Naming Test. Pro-ed; 2001.

[45] Piguet O, Hodges JR. Behavioural-variant frontotemporal dementia: an update. Demen Neuropsychol. 2013;7(1):10. 10.1590/S1980-57642013DN70100003 PMC5619539 29213814

[46] Freeman RQ, Giovannetti T, Lamar M, et al. Visuoconstructional problems in dementia: contribution of executive systems functions. Neuropsychology. 2000;14(3):415-426. 10928745 10.1037//0894-4105.14.3.415

[47] Kinnunen KM, Cash DM, Poole T, et al. Presymptomatic atrophy in autosomal dominant Alzheimer's disease: a serial magnetic resonance imaging study. Alzheimers Dement. 2018;14(1):43-53. 28738187 10.1016/j.jalz.2017.06.2268 PMC5751893

